# Time to develop adverse drug reactions and associated factors among children HIV positive patients on antiretroviral treatment in North West Amhara Specialized Hospitals: Retrospective cohort study, 2022

**DOI:** 10.1002/hsr2.1933

**Published:** 2024-02-25

**Authors:** Mequanente Dagnaw, Meera Indracanti, Bisrat Misganaw Geremew, Esubalew Asmare Mekonnen, Muluken Tekle, Mulu Muche, Dagnachew Wassie Gelaw, Bogale Damtew Amera

**Affiliations:** ^1^ Department of Epidemiology, Institute of Public Health, Institute of Biotechnology, Department of Medical Biotechnology University of Gondar Gondar Ethiopia; ^2^ Department of Medical Biotechnology, School of Allied Health Sciences Malla Reddy University Hyderabad Telangana India; ^3^ Department of Epidemiology and Biostatics, Institute of Public Health University of Gondar Gondar Ethiopia; ^4^ Department of Biology, College of Natural and Computational Sciences University of Gondar Gondar Ethiopia; ^5^ Department of Microbiology,Immunology and Veterinary Public Health, College of Veterinary Medicine Addis Ababa University Addis Ababa Ethiopia; ^6^ Department of Environmental Biotechnology, Institute of Biotechnology University of Gondar Gondar Ethiopia; ^7^ Department of Biotechnology, Institute of Biotechnology University of Gondar Gondar Ethiopia

**Keywords:** adverse drug reactions, Ethiopia, HIV/AIDS, time

## Abstract

**Introduction:**

Adverse drug reactions (ADRs) are harmful and unintended reactions to medicines given at standard doses through a proper route of administration for the purpose of prophylaxis, diagnosis, or treatment.

**Objective:**

The objective of this research paper was to assess median time to development of ADRs and associated factors among children HIV positive patients on antiretroviral treatment (ART) in North West Amhara Specialized Hospitals.

**Methods:**

The adverse drug effect survival time was estimated using the Kaplan–Meier survival method and log‐rank test curves was applied for analyze “time‐to‐event” data. Cox regression model was used to identify the associated factors. Adjusted hazard ratios with their respective 95% confidence intervals (CIs) were estimated and a value of *p* less than 0.05 was used to declare the presence of a significant association.

**Result:**

The overall incidence of ADRs was 0.67 (95% CI: 3.74–4.44) per 10,000 person‐year observation, with a median of 57 months. Adults are presenting with opportunistic Infections (OIs) experiences, baseline CD4 < 200 cells/µL counts, 1e, tenofovir disoproxil fumarate–lamivudine–efavirenz ART regimen, bedridden baseline functional status, World Health Organization (WHO) clinical stage II and III were notably associated with the incidence of ADRs development.

**Conclusion:**

ADRs were uncommon in this study. predictors, such as OIs experiences, a low CD4 count, ART regimen, an advanced WHO stage, and bedridden functional status were significantly associated with ADRs.

## 1 BACKGROUND

Human immunodeficiency virus/acquired immune deficiency syndrome (HIV/AIDS) infection disease remains the important cause of morbidity and mortality throughout the world.^1^ In a 2018 report, an assessed 37.9 million people globally were living with HIV, among these 54% of people living with HIV (PLHIV) reside in Eastern and Southern Africa.^2^ Sub‐Saharan Africa is the most maximumly affected area including Ethiopia ranking within the top 25 countries with the highest new HIV infection rates. In 2017 report 36.9 million in the world living with HIV/AIDS, and new infections have seen a reduction of 18% since 2010. But this rate of deterioration is not adequate for the goal of eradication of AIDS by 2030. Only 21.7 million people infected with HIV have access to antiretroviral (ARV) therapy, with the rest at risk of the potential difficulties of HIV infection.^3^

Since antiretroviral treatment (ART) introduction, the therapy has considerably reduced the morbidity and mortality caused by HIV infection.^4^ Anti‐HIV drugs act by preventing viral multiplication, thereby improving the immune system's response and decreasing the risk of transmission to sexual partners and children.^5^ Unfortunately, alongside these gains, ARVs, like many other administered drugs, are reported to be associated with adverse drug reactions (ADRs). An ADR is any noxious, unintended, and undesired effect of a drug, which occurs at normal doses used in humans.^6^ The outcome of ADRs is observed in both adults and children.^7^ Millions of eligible HIV‐infected patients have access to life‐prolonging ARV drugs. This has led to an appreciable decrease in HIV‐related morbidity and mortality.^7^ Like most chronically administered drugs, ARVs have documented toxicities and adverse effects. ADRs range from mild to life‐threatening with short‐ and long‐term effects, however little is known about the ADRs of ARVs in many HIV programs in the public health sector of developing countries.^8^ The spectrum of adverse effects associated with ARVs may vary between developed and developing countries.^9^ Variance in psychological and socioeconomic support of HIV positive patients in the public health sector of developing. Countries coupled with co‐morbidities make monitoring ADRs to ARV a necessity. Studies on the incidence of ADR from developing and developed countries have reported the incidence of ADR among patients on ARVs to range between 11% and 35.9%^10^, ^11^ with an incidence as high as 54%^12^ in the presence of opportunistic infection (OI). Incidence of severe ADR has been reported to be as high as 10% [6] with a study observing an incidence rate of 8 per 100 person‐years (PY).^7^ The long‐term effects of ARTs are largely unknown but ongoing research provides insights into some ADRs of ARV.^13^ These include peripheral neuropathy and lipodystrophy associated with stavudine,^7^ anemia associated with zidovudine (AZT),^14^, ^15^ and nevirapine (NVP)‐based hepatotoxicity and rash.^15^, ^16^ Incidence of hepatotoxicity was observed to be 16% and 8% for patients on NVP and efavirenz (EFV), respectively^17^ while the incidence of anemia ranged from 3% to 12% among patients on AZT in developing countries including Ethiopia.^9^

There is substantial evidence linking treatment success to adherence to ARVs.^18^ However, treatment adherence is closely linked to ADRs.^13^, ^18^ It is thus imperative that clinicians clearly understand ADRs, readily recognize them in patients, and manage them effectively. Most studies on ADRs are clinical trials and represent a select group of cohorts; however, studies of large cohorts of unselected patients are more suited to inform on the situation of ADRs in actual clinical practice of the public health sector.

Many studies have been carried out around the world, and the results from these studies have shown that the incidence of ADRs among patients on ART ranged between 4.3% and 90%.^19^ Some factors including the age of the patient, gender, ART regimen, duration of treatment, OI prophylaxis, World Health Organization's (WHO) clinical stage, disease biomarkers, and body mass index (BMI) were shown to be associated with the development of ADR.^19^ The weakness of the immune system due to the infection and the complexity of anti‐HIV drugs can also affect the occurrence of ADRs. Previous studies have described associations between ART use and a large spectrum of ADRs. These included lipodystrophy, abdominal pains, fatigue, nausea and vomiting, diarrhea, hepatotoxicity, hypersensitivity syndrome reactions (rashes), central nervous system adverse events, and pancreas and kidney toxicities.^20^ The severity of ADRs ranges from mild to life‐threatening and may occur following a single dose or prolonged administration of the medicine. The combination of two or more medicines may also aggravate the condition. Drug toxicities can add to the complexity of the disease's management by affecting patient compliance with the treatment. The plausible resulting consequences could be the poor response to treatment associated with higher costs to the public health system.^21^ Generally, about 6% of all admissions into medical hospital wards are due to ADRs. Some pieces of evidence have shown that, in up to one‐quarter of patients, there was a modification of the initial ART regimen due to ADRs.^22^ Therefore, drug adverse effect continues to cause substantial morbidity and mortality in patients with HIV‐1 infection despite the use of ART. Even though one study such as in Bahir Dar City, Hospital has been conducted on the time to develop drug adverse effects of individual among HIV‐infected adult patients on ART in Ethiopia but information about the time to develop of drug adverse effects and its associated factors among children is scarce in the University of Gondar comprehensive specialized referral hospital, North‐West Ethiopia. Therefore, this study will assess the time to develop drug adverse effects and identify associated predictors in patients taking ART drugs at the University of Gondar comprehensive specialized referral hospital, in Northwest Ethiopia.

## 2 METHODS

### 2.1 Study design and period

An institution‐based retrospective follow‐up study was conducted on children living with HIV on highly active antiretroviral treatment (HAART) attending chronic HIV care clinics at selected Compressive Specialized Hospitals, Amhara regional state, north‐west Ethiopia, January 11, 2017, to January 10, 2022.

### 2.2 Study setting

The study area will be three selected referral hospitals like University of Gondar Compressive Specialized Hospital. The University of Gondar Compressive Specialized Hospital is located in Northwest Ethiopia serving approximately 5 million populations. A variety of diseases including both communicable and noncommunicable are reported in the catchment population. Health services units include outpatients' clinics, maternity clinics, emergency ward, adult in‐patients, pediatrics in‐patients, community clinics, and Laboratory services. The hospital has 518 beds and about 350–400 patients visit each day and of these, about 100–120 patients visit in the emergency unit. The hospital has four emergency suites with a triage unit for distribution. It is staffed by about 270 nurses and 150 physicians.^23^

The hospital has been providing HIV‐care and ART follow‐up services since 2005 E.C. Currently, the ART clinic of this hospital has 5481 ART follower patients among this 1138 ART follower HIV‐infected children, 1 medical doctor, 10 nurses, 6 data clerks, 3 porter, 2 cleaner, 6 case managers, and 7 ART education adherence counselors. The hospital uses standardized ART monitoring and evaluation tools adapted from the Ethiopian national comprehensive HIV care and treatment guidelines.

Bahir Dar city is the capital city of Amhara National, Regional State,^24^ and is located about 570 km northwest of Addis Ababa. The city is bordered by Lake Tana in the north, South Gondar in the east, and by West Gojjam in the south and west. The city has 9 subcities and 12 rural kebeles with a total population of 308,887.^24^ The city has 1 university, 2 referral hospitals, 1 district hospital, 10 health centers, and 2 private hospitals. The number of healthcare workers in both public and private facilities is 823. The study was conducted two public hospitals (one referral and one district) and two private hospitals.

Debre Tabor Specialized Hospital: Location from Addis Ababa approximately 669 km from A.A. Serving population approximately 4,000,000 in number, types of health services units: mainly include outpatients' clinics, maternity clinics, emergency ward, adult in‐patients, pediatrics in‐patients, laboratory services, explain, surgical, radiology, orthopedics more than 15 services, number of hospital beds 308, average numbers of patients visited each day approximately 550, number of staffs working in the hospital 529. Number and professions of technical staffs: physicians 57, nurses 134, laboratories 25, pharmacies 30, environmental health 2, others 69, supportive employees, 212, ART clinics, types of services in ART clinics, people living with HIV, registered, currently on follow‐up 2137, number of, males 834 females 1260, adults 2094, children (≤15 years), males 25 females 18 (make it near 100 b/c he counts only children on follow‐up). Number and professions of technical staffs in ART clinics, physicians 2, nurses, 4, laboratories 2, pharmacies 2, others 6.

### 2.3 Source population and study population

#### 2.3.1 Sources population

The source population for this study was all HIV‐infected children (aged ≤15 years) ever initiated on ART at the University of Gondar compressive specialized Referral Hospital and Debre Tabor Specialized Hospital.

#### 2.3.2 Study population

All patients with HIV/AIDS aged ≤15 years enrolled for HIV care at the University of Gondar compressive specialized Referral Hospital and Debre Tabor Specialized Hospital from January 1, 2017 to March 31, 2022 participated were the study populations.

### 2.4 Inclusion and exclusion criteria

#### 2.4.1 Inclusion criteria

All children living with HIV aged ≤15 years within the study population including those HIV‐infected children who started on ART and missed the outcome of interest between January 2017 and March 31, 2022, were included in the study and followed for 5 years, until March 31, 2022. This period was selected to have the nearest 5‐year follow‐up study period.

#### 2.4.2 Exclusion criteria

All children living with HIV aged ≤15 years adults who had missing data such as incomplete baseline information which affects outcome variable and follow‐up data, missing the date of enrollment, missing the date of occurrence of drug adverse effect, and/or who had adverse drug effect at the time of ART initiation were excluded from the study.

### 2.5 Sample size and sampling procedures

#### 2.5.1 Sample size

In this study, sample size was determined by using single population proportion formula by taking the proportion 17.3% from a previous study, which was done on adult HIV patients in Ethiopia.^22^ With the following assumptions under consideration, desired degree of precision of 3%, and 95% of confidence interval it provides 602 and with the consideration of 15% withdrawal it gives 692.

#### 2.5.2 Sampling procedures

This study was carried out in North West Amhara Specialized Hospitals that provide ART service during the specified study period, University of Gondar Compressive Specialized Hospital, Felege Hiwot Comprehensive Specialized Hospital, and Debre Tabor Specialized Hospital. Therefore, the total sample size was proportionately allocated for these three selected specialized Hospitals based on the number of the study population. A total of 692 medical cards were reviewed, of which 7 were excluded due to ineligibility and incompleteness (more than 15% of variables were incomplete). Thus, medical records of HIV patients who fulfilled the inclusion criteria were included with the above‐mentioned public health facilities until the calculated sample size was attained. A simple random sampling technique with computer‐generated random numbers was used and medical records of those HIV patients who start ART between January 11, 2017, to January 10, 2022, were considered as a sample frame.

### 2.6 Study variables

The two outcome variables considered for the study were the survival outcome which was the time to develop ADRs measured from the time shortly after the start of HAART (within 12 weeks), occurring >3 months after initiation of HAART and CD4 count >200 cells/mm^3^ and occur in patients with virology and immunologic failure on HAART of co‐infection from HIV infectious disease individual or censored in a month. However, the time to develop ADRs was censored for those co‐infected patients who lost the follow, transferred to another Hospital, and was not developed ADRs at first January 2017 (at the beginning of the study). Hence, the longitudinal outcome was virologic load was conducted to measure the counts per mm^3^ of blood which was measured within 6 months interval and acted as a biomarker for co‐infected patients.

#### 2.6.1 Dependent variable

The dependent variable for this study was the median time to develop ADRs during follow‐up periods.

#### 2.6.2 Independent variables

The independent variables included: Sociodemographic characteristics (i.e., sex, age, residence, religion, family size, nutritional support, and housing status); clinical predictors (i.e., baseline CD4 count, viral load, past ADR status, WHO clinical stage, baseline weight, functional status, and past ADR and opportunistic illness); and ART and other medication‐related predictors (i.e., eligibility criteria, date ART started, type of baseline ART regimen, original regimen taken when the patient first starts ART, Is there regimen change during the follow‐up, if yes reason for regimen the patient change if any, date of switching to second line regimen, If switched determined by and Adherence).

### 2.7 Operational definitions

#### 2.7.1 ADRs

A response to a medicine that is noxious and unintended, and which occurs at doses normally used in humans.^25^

#### 2.7.2 Event

Any untoward medical occurrence that may present during treatment with a pharmaceutical product, but which does not necessarily have a causal relationship with this treatment.

#### 2.7.3 Censored

The nonrecurrence of any OIs, lost to follow‐up and transferred out in study participants during the follow‐up period.

#### 2.7.4 Lost to follow up

If PLHIV on HIV care not seen for equal to or more than 1 month as recorded by ART clinic personnel;

#### 2.7.5 Transferred out

If PLHIV on HIV care in one health institution shifts to another health institution.

#### 2.7.6 Good adherence

if adherent ≥95%, that is the percentage of missed dose is <2 doses of 30 doses or <3 dose of 60 doses) as documented by ART health personnel.

#### 2.7.7 Fair adherence

if adherent 85%–94%, that is, the percentage of missed doses is 3–5 doses of 30 doses or 3–9 doses of 60 doses) as documented by ART health personnel.

#### 2.7.8 Poor adherence

if adherent <85%, that is, the percentage of missed doses is ≥3 doses of 30 doses or >9 doses of 60 doses) as documented by ART health personnel.

### 2.8 Data collection tools and procedures

A pretested structured data collection checklist adapted from Ethiopia Ministry of Health 2020 guideline was used to extract routinely recorded data from children living with HIV who initiated ART treatment in the Hospitals. All charts containing detailed data, including the baseline CD4 count, viral load, hemoglobin, and repeated measures of CD4 cell counts almost every 6 months were reviewed. Similarly, other characteristics, like sociodemographic (age and sex), clinical (baseline WHO clinical stage, virologic load, baseline functional status), residence, and baseline regimen together with others were also collected from the registration book of ART users. Three health professionals (BSc Nurses) trained for 2 days retrieved the data. The principal investigator and the supervisor closely monitored the process throughout the data collection period and made due corrections.

### 2.9 Quality assurance

To assure data quality, a data extraction checklist was carefully adopted from a standardized ART intake and follow‐up forms, nurses who currently work in the ART clinic and who took ART training was recruited as data collectors, and a 2‐day training was given for both data collectors and supervisors, completeness of the recorded variables was double checked by taking some randomly selected cards, and the supervisor, as well as principal investigators, carefully monitored the entire data collection process. Subsequently, all relevant data were retrieved by reviewing HIV‐infected children's cards. The occurrence of DAR during data extraction was ascertained by reviewing the health professionals' reporting on patient charts. Any laboratory tests obtained at the time of ART initiation were considered baseline data. However, if laboratory tests were not done during ART initiation, any lab tests done within a month of ART initiation were considered as the baseline.

### 2.10 Data processing and analysis

The data was edited and cleaned using the statistical software Epi data 4.6. version by cross‐checking missing collected data. The data were checked for inconsistencies, coding errors, completeness, accuracy, clarity, and missing values. The edited and cleaned data were analyzed using the statistical software called STATA version 16. The data was processed to generate the survival time to develop DAR and the censoring indicator. Summary measures such as texts, tables, graphs count, percentages, medians, interquartiles (IQRs), means, and SD were used to show frequencies of sociodemographic, clinical, and other variables calculated and presented. The survival time until the patient's time to develop DAR was also described. Incidence rate DAR by each characteristic of patients, including an overall incidence rate was determined. The frailty model was employed to determine the adjusted effect of available variables on the event of interest after controlling for variables not included in the model. Before using the survival regression models, the assumption of proportional hazard was checked using the log–log technique. Weibull, exponential, and lognormal distributions were used alternatively to get the fit of the baseline hazard. The Kaplan–Meier survival method and log‐rank test curves were applied to analyze “time‐to‐event” data and the test compares the entire survival experience between groups and can be thought of as a test of whether the survival curves are identical (overlapping) or not, respectively. Cox proportional hazards assumption was checked both graphically^26^ and using the Schoenfeld residuals test. Model selection was done using Akaike information criteria (AIC) and Bayesian information criteria (BIC). The parametrical survival analysis, and different distributions, were compared and used to identify predictors of time to develop DAR. Model goodness of fit was checked using the Cox–Snell residual test. Adjusted hazard ratios (AHRs) with their respective 95% CIs were estimated and a value of *p* less than 0.05 was used to declare the presence of a significant association.^27^, ^28^

## 3 RESULT

### 3.1 Sociodemographic characteristics of HIV/AIDS patients on ART treatment

A total of 685 HIV‐infected children on ART were included in the final analysis. More than half, 345 (51%), of the study participants were females. By the time of enrollment into ART care, 423 (62%) of the participants were between 1 and 10 years of age. The majority of the study participants were orthodox for 286 (42%) of the total sample. Regarding educational status, 282 (41%) of patients had secondary education (Table 1).

**Table 1:** Sociodemographic characteristics/variables of HIV/AIDS patients on ART treatment at the University of Gondar Compressive Specialized Hospital, Felege Hiwot Comprehensive Specialized Hospital and Debre tabor Specialized Hospital, January 11, 2017, to January 10, 2022 (*n* = 685).

Variables	Category	Frequency (*N*)	Percentage (%)
Sex	Female	345	0.51
	Male	340	0.49
Age	1–10	423	0.62
	11–15	262	0.38
Religion	Orthodox	286	0.42
	Muslim	210	0.31
	Protestant	189	0.28
Education	No education	158	0.23
	Elementary	245	0.36
	Secondary	282	0.41
Occupation	Student	527	0.77
	Daily labor	158	0.23
Residence	Urban	565	0.82
	Rural	120	0.18
Household	1–2	187	0.27
	3–5	435	0.64
	6–8	63	0.91
Caregiver	Yes	535	0.78
	No	150	0.22
Disclosure	Disclosed	645	0.94
	Not disclosed	40	0.06
Parent HIV status	Positive	367	0.54
	Unknown	318	0.46

Abbreviations: AIDS, acquired immunodeficiency syndrome; ART, antiretroviral therapy; HIV, human immunodeficiency virus.

### 3.2 Clinical, immunological, and treatment characteristics of HIV/AIDS patients on ART treatment

Among the study subjects, 438 (64%) had baseline CD4 count >200 cells/μL. Based on the baseline WHO clinical stage, 379 (55%) of patients were stage I and 267 (39%) stage II, respectively. Mostly 369 (54%) of the participants had a working functional status and 469 (68%) of the study participants were taken a first‐line regimen. The majority of the study participants had normal BMI accounting for 468 (68%) (Table 2).

**Table 2:** Clinical characteristics/variables of HIV/AIDS patients on ART treatment at the University of Gondar Compressive Specialized Hospital, Felege Hiwot Comprehensive Specialized Hospital, and Debre tabor Specialized Hospital, January 11, 2017, to January 10, 2022 (*n* = 685).

Variables	Category	Frequency (*N*)	Percentage (%)
ADS	Yes	212	0.31
	No	473	0.69
Prophylaxis	CPT	337	0.49
	INH	245	0.36
	Fluconazole	103	0.15
CD4 count	≤200 cell/µL	247	0.36
	>200 cell/µL	438	0.64
Hgb	≤12	267	0.39
	13–17.5	418	0.61
TB co‐infection	Yes	169	0.25
	No	516	0.75
BMI	Normal	468	0.68
	Mild	129	0.19
	Moderate	88	0.13
Functional status	Bedridden	196	0.29
	Ambulatory	120	0.18
	Working	369	0.54
WHO clinical stage	Stage I	379	0.55
	Stage II	267	0.39
	Stage III	39	0.06
Initial ART regimen	First‐line regimen	469	0.68
	Second‐line regimen	179	0.26
	Third‐line regimen	37	0.05
Drug adherence	Good	342	0.50
	Fair	301	0.44
	Not recorded	42	0.06

Abbreviations: AIDS, acquired immunodeficiency syndrome; ART, antiretroviral therapy; BMI, body mass index; Hb, hemoglobin; HIV, human immunodeficiency virus; TB, tuberculosis; WHO, World Health Organization.

### 3.3 Adverse drug reaction incidence from HIV/AIDS patients on ART treatment

During the 6‐year retrospective follow‐up, 153 (22.34%) had developed ADRs, 134 (19.56%) died, 230 (33.58%) were transferred out to another health facility, 101 (14.74%) were lost to follow‐up, and 67 (9.78%) were on follow up without experiencing ADRs. The proportions of ADR are 0.28 (0.25, 0.32). Patients were followed for a minimum of 1 month and a maximum of 80 months with a total of 545,845 person‐months of observation and the median observation time was 56 months with IQR: 57 months. Overall, there were 67 incidents of ADR reported during 545,845 person‐months of observation, or 544,771 person‐years. And the incidence density rate was 0.67/1000 person's month (CI: 0.60–0.74/1000 person's month).

### 3.4 Predictors of ADR

**Figure 1:**
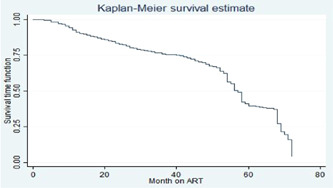
The Kaplan–Meier curve showing a cumulative probability of adverse drug reactions among human immunodeficiency virus/acquired immune deficiency syndrome patients on antiretroviral therapy treatment.

On the bivariable Cox regression, covariates such as types of health facility, sex, age category, educational status, residence, occupation, ART regimen, functional status, weight for age, WHO clinical stage at ART initiation, BMI at ART initiation, OIs, CD4 count at initiation of ART, height for age, types of regimens, developmental status, and cotrimoxazole preventive were significant at ≤0.25 *p* value (Figure 1).

### 3.5 Predictors of ADR among HIV/AIDS patients on ART treatment

**Figure 2:**
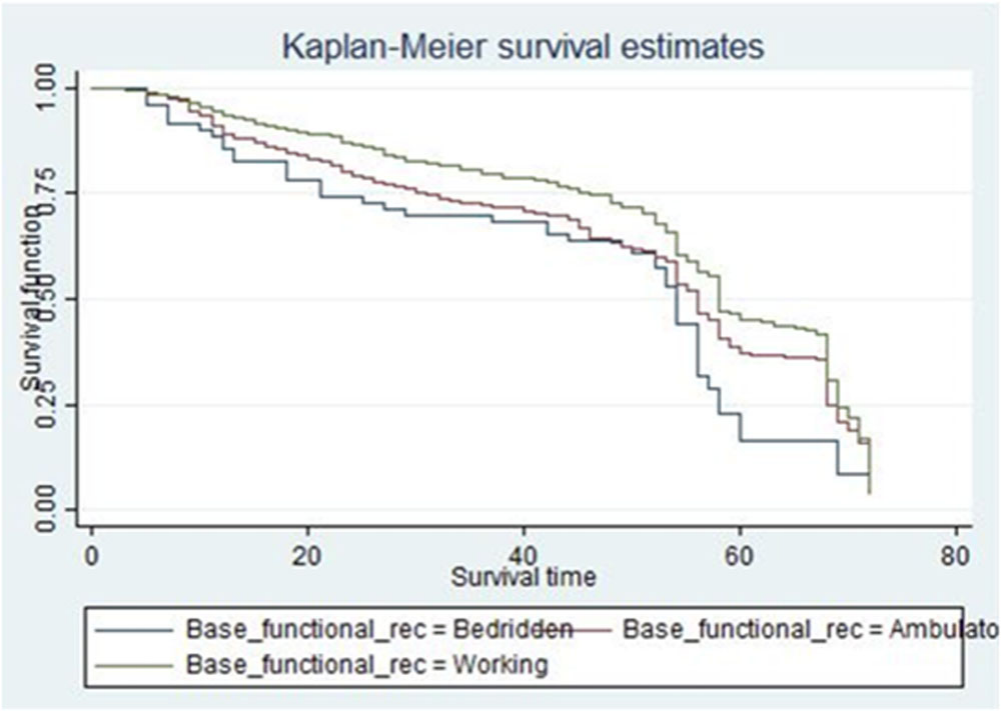
The Kaplan–Meier survival curves for covariates curve showing baseline functional status of human immunodeficiency virus/acquired immune deficiency syndrome patients on antiretroviral therapy treatment.

The graph of the estimate of the overall Kaplan–Meier function is given in Figure 1. Separate graphs of the estimates of the Kaplan–Meier functions for some of the categorical variables. The upper curve in the figure indicates that the particular group experiences more survival time than the one below. To investigate if there was a significant difference between the ADR across different groups of baseline functional status this figure shows that the baseline working functional status group had more survival time as compared to that of baseline bed redden functional status group one (Figure 2).

**Figure 3:**
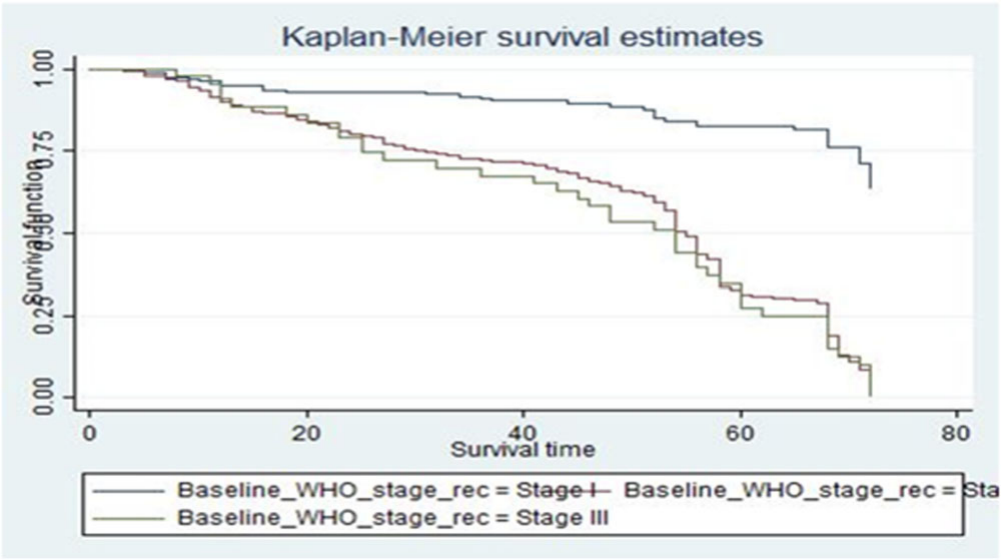
The Kaplan–Meier survival curves for covariates curve showing the World Health Organization (WHO) clinical stage of human immunodeficiency virus/acquired immune deficiency syndrome patients on antiretroviral therapy treatment.

HIV/AIDS patients who have baseline WHO clinical stage I at the start of ART treatment had longer survival experience than HIV/AIDS patients with baseline WHO clinical stage III (Figure 3), which is supported by log‐rank test (log‐rank *χ* ^2^(1) = 105.67, *p* = 0.00 (Table 3).

**Table 3:** The log‐rank test results for categorical variables in OIs HIV/AIDS patients on ART treatment in the University of Gondar Compressive Specialized Hospital, January 11, 2017, to January 10, 2022 (*n* = 685).

Variables	*Df*	*χ* ^2^	*p* Value
Sex	1	0.01	0.76
Age	5	4.90	0.43
Education	3	26.02	0.00
Occupation	4	9.66	0.04
Prophylaxis	2	34.73	0.00
CD4 count	2	34.73	0.00
Functional status	2	18.19	0.00
WHO clinical stage	3	108.77	0.00
ART side effect	1	5.78	0.02

Abbreviations: AIDS, acquired immunodeficiency syndrome; ART, antiretroviral therapy; HIV, human immunodeficiency virus; OIs, opportunistic infections; WHO, World Health Organization.

### 3.6 Assessing the proportional hazard assumption

To fit a model, we have to assess some requirements of the model which means the model should be assessed whether it describes our data well or not. In this instance, the primary objective was to validate the proportional hazard assumption and to determine the model's overall goodness of fit. The proportional hazard assumption states that the study subjects' risk of failure must remain constant regardless of how long they are followed.

**Figure 4:**
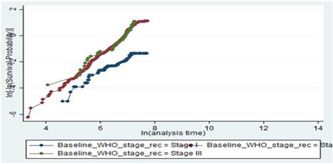
Plot of log (−log (survival probability)) versus log (survival time) by baseline World Health Organization (WHO) clinical stage for type human immunodeficiency virus/acquired immune deficiency syndrome patients on antiretroviral therapy treatment.

The log‐log plot (survival probability) versus the log of survival time was done for different categories of predictor variables. As observed from the plot of three categories of baseline WHO clinical stage, the three lines were nearly parallel which means that the proportional hazard assumption was valid. Plots of scaled Schoenfeld residuals show randomness. Moreover, the smoothed curve is an approximately horizontal line, so the assumption of proportional hazards was also satisfied repeatedly using this method (Figure 4).

The global test of the proportional hazards assumption based on the Schoenfeld residuals was also done, and it was found that all covariates and the full model satisfy the proportional hazard assumption (*χ* ^2^ = 16.32, *p* = 0.2999) in the process of model development.

### 3.7 Comparative analysis of models

After confirming the proportional hazard assumption, semi‐parametric and parametric proportional hazard models were fitted to estimate the survival incidence of OIs and to identify predictors in HIV/AIDS patients. Using information criterion (AIC, BIC) and log‐likelihood results, the most sparsity model was chosen. Based on all three comparison techniques used, the Gompertz regression model (AIC = 1207.696, BIC = 133.147, log likelihood = −576.8479) was more efficient than Cox‐PH and other parametric models. Interpretations and conclusions were thus based on the Gompertz model (Table 4).

**Table 4:** Summary of model comparison between semi‐Cox proportional hazard models and parametric Cox‐regression models using AIC, BIC, and log‐likelihood.

Methods	Comparison models type					
	Cox PH	Exponential	Gompertz	Loglogistic	Weibull	Lognormal
Log likelihood	−2954.238	−744.16944	−576.8479	−671.793	−598.20	−694.05
AIC	5958	1540.339	1207.696	1391.58	1250.4	1443.87
BIC	607.784	1659.218	133.147	1501.32	1373.8	1567.32

Abbreviations: AIC, Akaike information criteria; BIC, Bayesian information criteria; PH, proportional hazard.

After fitting a univariate Gompertz proportional hazard model, all the predictor variables were found to have *p* < 0.25; after this, a multivariable model was fitted, and covariates like baseline OIs, CD4 count, 1e, tenofovir disoproxil fumarate–lamivudine–EFV (TDF‐3TC‐EFV) ART regimen, baseline bedridden functional status and baseline WHO clinical stage II and III were found to be significant predictors for incidence of ADRs among HIV/AIDS patients at 5% level of significance.

The hazard of developing ADRs is increased by 96% among HIV/AIDS patients who have experienced OIs than those patients who have no OIs (AHR = 2.96 [2.77, 3.20]).

The hazard of developing ADRs is increased by 41% among HIV/AIDS patients who have baseline CD4 count ≤ 200 cell/μL than those patients who have baseline CD4 count > 200 cell/μL (AHR = 1.41 [1.18, 1.69]).

The hazard of developing ADRs among HIV/AIDS patients with bedridden baseline functional status increases by 35% compared to patients with working baseline functional status (AHR = 1.35 [1.01, 1.82]).

The hazard of developing ADRs among HIV/AIDS patients at baseline WHO clinical stage II is 5.87 times higher than patients at baseline WHO clinical stage I (AHR = 5.87 [3.97, 8.69]).

The hazard of developing ADRs among HIV/AIDS patients at baseline WHO clinical stage III is 5.85 times higher than patients at baseline WHO clinical stage I status HIV/AIDS patients (AHR = 5.85 [3.55, 9.65]) (Table 5).

**Table 5:** Cox regression analysis between different predictor variables and time to the development of ADRs among adult HIV‐positive patients on HAART regimen at University of Gondar Compressive Specialized Hospital, Felege Hiwot Comprehensive Specialized Hospital, and Debre Tabor Specialized Hospital, January 11, 2017, to January 10, 2022 (*n* = 685).

Covariates	ADRs		CHR (95% CI)	AHR (95% CI)	*p* Value
	Yes	No			
Education					
No education	85	15	1.56 (1.19, 2.03)	1.28 (0.98, 1.69)	0.073
Secondary	200	83	0.99 (0.79, 1.25)	0.89 (0.71, 1.13)	
OIs					
No	400	112	1	1	
Yes	115	58	0.76 (0.61, 1.81)	2.96 (2.77, 3.20)	0.021
CD4 count					
≤200	231	20	1.98 (1.66, 2.36)	1.41 (1.18, 1.69)	0.000
(>200)	314	120	1	1	
ART regimen					
1c, AZT‐3TC‐NVP	17	13	1	1	
1d, AZT‐3TC‐EFV	33	63	2.03 (1.03, 4.00)	0.68 (0.29, 1.62)	0.092
1e, TDF‐3TC‐EFV	422	107	2.26 (1.71, 2.97)	3.04 (1.77, 5.22)	0.001
1f, TDF‐3TC‐NVP	21	9	1.20 (0.74, 1.93)	1.28 (0.97, 1. 70)	0.084
Functional status					
Bedridden	59	10	1.50 (1.13, 2.00)	1.35 (1.01, 1.82)	0.040
Ambulatory	203	44	1.31 (1.09, 1.57)	1.16 (0.96, 1.41)	0.120
Working	253	116	1	1	
WHO clinical stage					
Stage I	30	126	1	1	
Stage II	443	43	9.13 (6.20, 13.44)	5.87 (3.97, 8.69)	0.000
Stage III	42	1	9.82 (6.02, 16.01)	5.85 (3.55, 9.65)	0.000

*Note*: LR test *χ* ^2^(25) = 286.34 Prob > *χ* ^2^ = 0.0000.

Abbreviations: 3TC, lamivudine; ADR, adverse drug reaction; AHR, adjusted hazard ratio; ART, antiretroviral treatment; AZT, zidovudine; CHR, crude hazard ratio; CI, confidence interval; EFV, efavirenz; HAART, highly active antiretroviral treatment; HIV, human immunodeficiency virus; LR, likelihood ratio; NVP, nevirapine; OI, opportunistic infection; TDF, tenofovir disoproxil fumarate; WHO, World Health Organization.

The shape parameter gamma was found to be 0.02 (95% CI: 0.023, 0.029) which is positive. This indicates that the hazard of OIs increases exponentially with time.

### 3.8 The goodness of fit test

The Cox–Snell residuals (together with their Nelson–Aalen cumulative hazard function) had been obtained from fitting using the exponential, Weibull, Gompertz, and lognormal log‐logistic models to our data. It can be seen that the plot of the Nelson–Aalen cumulative hazard function against Cox–Snell residuals was closest to 45° relatively straight lines through the origin for the Gompertz model when compared to Weibull and others. This suggests that the Gompertz model provided the best fit for our data set.

## 4 DISCUSSION

This study mainly investigated the incidence and predictors of ADRs among HIV/AIDS patients on ART treatment at the University of Gondar Compressive Specialized Hospital, Felege Hiwot Comprehensive Specialized Hospital, and Debre Tabor Specialized Hospital, Ethiopia. Many other studies reported different risk predictors for time to develop ADRs; our study also assessed the patients' sociodemographic, clinical, and treatment characteristics based on the records from their medical follow‐up charts. As a result, predictors like OIs, baseline CD4 count, ART regimen, baseline functional status, and baseline WHO clinical stages II and III were significant predictors of the time to develop ADRs. This finding is similar to the study done by Arba‐Minch,^26^ Hossana,^29^ Mekelle,^30^ and Debre Markos,^31^ which reported the WHO clinical stage and CD4 count variables as risk predictors for the development of ADR. Out of 685 patients in the cohort study, 28% (CI: 25–32) developed different ADRs. This study is comparable to one conducted in Nigist Elleni Mohamed Memorial Comprehensive Specialized Hospital, Southern Ethiopia. This might be due to the difference in patients' awareness,follow‐up time, and ART service difference in this two‐study area.

During the study period, the overall incidence rate of ADRs after a median follow‐up time of 57 months was 0.67/1000 person's month (CI: 0.60–0.74/1000 person's month) observation. This result was a little bit lower than the study done in Africa,^32^ which showed the cumulative incidence to be 53.6 per 100 people per year, in Arba Minch, which showed 23.9/100 PY (95% CI: 18.3, 29.5).^26^, ^33^

In our study, the HIV/AIDS patients who had OIs experienced increase by 96% than to HIV/AIDS patients who had no OIs experience (AHR = 2.96 [2.77, 3.20]). This finding is similar to other research which is done in Aksum St Mary General Hospital in Tigray regional state, northern Ethiopia.^34^ The finding proves that the management of OIs using different drugs in addition to the ART regimen leads to the development of adverse drug reaction.^35^

In our study, HIV/AIDS patients who had baseline CD4 count ≤200 cell/μL increased by 41% more than among HIV/AIDS patients who have baseline CD4 count > 200 cell/μL (AHR = 1.41 [1.18, 1.69]). This finding is similar to other research which is done in Nigist Elleni Mohamed Memorial Comprehensive Specialized Hospital, Southern Ethiopia.^29^ The finding proves that CD4 cells play a central role in activating both humoral and cellular immune responses of patients' bodies tofight against infection. Hence, a low CD4 count increases susceptibility to ADRs more than the higher one.^36^

In our study, HIV/AIDS patients who had 1e, TDF‐3TC‐EFV ART regimen is increased by 4% than among HIV/AIDS patients who have 1c, AZT‐3TC‐NVP, 1d, AZT‐3TC‐EF, 1f, TDF‐3TC‐NVP ART regimen (AHR = 0.28 [0.25, 0.32]). This finding is also similar to other research which is done in Arba Minch General Hospital, Arba Minch, and Secha Health Center, Southern Ethiopia.^37^ This study found that AZT and NVP‐based regimen was significantly more toxic than tenofovir and efaverinz‐based regimens. This finding was supported by a study done in Uganda in 2013 in which ART‐related ADRs were higher in NVP containing regimen compared to efaverinz and also a study in Ethiopia at Hiwot Fana hospital, in which TDF‐based regimens were less toxic compared to AZT‐containing regimens.^38^, ^39^

In our study, HIV/AIDS patients with bedridden baseline functional status are increased by 35% compared to patients with working baseline functional status (AHR = 1.35 [1.01, 1.82]). This could be due to restrictions from physical activities and the inability to perform daily tasks, which, indirectly, compromise the immune system of the patients.^40^ This finding is also similar to other research which is done in Nigist Elleni Mohamed Memorial Comprehensive Specialized Hospital, Southern Ethiopia.^29^

In this study, the advanced stage of baseline WHO clinical stages II, and III was found to increase the risk of ADRs. The hazard of developing ADRs among HIV/AIDS patients on baseline WHO clinical stage II is 5.87 times higher than patients at baseline WHO clinical stage I (AHR = 5.87 (3.97, 8.69) and the hazard of developing ADRs among HIV/AIDS patients at baseline WHO clinical stage III is 5.85 times higher than the hazard of patients at baseline WHO clinical stage I status of HIV/AIDS patients, (AHR = 5.85 [3.55, 9.65]). This finding is similar to other studies in Ethiopia^29^ and in Nigist Elleni Mohamed Memorial Comprehensive Specialized Hospital, Southern Ethiopia.^37^ Often advanced baseline WHO clinical stages exhibit severe immune deficiency when the stage becomes more advanced, and the occurrence and recurrence of ADRs also increase. The most serious and life‐threatening ADRs are more common among HIV‐infected people with stage II and IV.^41^

The clinical importance of this study was to provide information for health professionals, researchers, policymakers, and patients about predictors that are associated with the risk of ADR development during ART follow‐uptime and to act on them to minimize the risk and maximize their efforts on prevention of having the problem and also its public health importance of this study is to prevent economic loss associated with the infections and its complications as a result of cost investment to prevention, treatment and control mechanisms.

## 5 LIMITATIONS OF THE STUDY

Since this study is a retrospective follow‐up study, using the patients' baseline sociodemographic, clinical, and treatment characteristics, there may be a change of these predictors (change of exposure predictors) after a time. Additionally, this study used secondary data, as robust data on some potentially significant predictors, such as behavior, distance to the hospital, and monthly income, were not available in‐patient charts.

## 6 CONCLUSION

The incidence rate of ADRs among HIV‐positive people treated at the University of Gondar Compressive Specialized Hospital, Felege Hiwot Comprehensive Specialized Hospital, and Debre Tabor Specialized Hospital, Ethiopia, ART Clinic were relatively lower than other study reports. More importantly, patient OIs experiences, baseline CD4 ≤ 200 cell/μL, 1e, TDF‐3TC‐EFV ART regimen, state‐of‐the‐art baseline WHO clinical stage, and baseline bedridden functional status were associated with a high risk of the incidence rate of ADRs development. I have corrected based on the suggestions: Since the current test and treat strategy is applied, it is better to strengthen the training of health professionals to overcome increased patient flow and to trace patients. Although ARV therapy increases the quality of life of HIV patients. It is better if appropriate measure is taken to support these at‐risk patients in terms of reminders, surveillance, and tracing mechanisms. It is better to take strict follow‐up, through telephoning or through social support groups and proper recording of their place of residence, those at‐risk populations such as younger age groups, daily laborers, ambulatory patients, and those taking AZT‐3TC‐NVP at the start of ART. Although this research is very useful in understanding predictors of ADRs it is also better to trace patients to investigate the exact reason for ADRs and what happens after a patient is lost to follow‐up.

## AUTHOR CONTRIBUTIONS

**Mequanente Dagnaw**: Conceptualization; data curation; formal analysis; methodology; software; writing—review and editing. **Meera Indracanti**: Conceptualization; data curation; supervision; writing—original draft. **Bisrat Misganaw Geremew**: Data curation; formal analysis. **Esubalew Asmare Mekonnen**: Data curation; formal analysis. **Muluken Tekel**: Data curation; formal analysis. **Mulu Muche**: Writing—review and editing. **Dagnachew Wassie Gelaw**: Data curation; formal analysis. **Bogale Damtew Amera**: Data curation; formal analysis.

## CONFLICT OF INTEREST STATEMENT

The authors declare no conflict of interest.

## ETHICS STATEMENT

The Public Health Institute, the College of Medicine, and Health Sciences, University of Gondar's Ethical Review Committee granted clearance and approval to conduct the research under the reference letter Reference No/IPH 22/07/2022. Because this study analyzed secondary data from patient charts, we were granted an informed consent waiver. To maintain confidentiality, the data collection tool did not include names or other personally identifiable information such as unique identification numbers.

## TRANSPARENCY STATEMENT

The lead author Mequanente Dagnaw affirms that this manuscript is an honest, accurate, and transparent account of the study being reported; that no important aspects of the study have been omitted; and that any discrepancies from the study as planned (and, if relevant, registered) have been explained.

## Data Availability

The corresponding author will have the right access to the data upon request.
